# Effectiveness of Chemotherapy on Long-Term Survival in a Case of Advanced Juvenile Hepatocellular Carcinoma Without Viral Hepatitis Infection

**DOI:** 10.7759/cureus.53278

**Published:** 2024-01-31

**Authors:** Masamichi Kimura, Koji Nishikawa, Jun Imamura, Kiminori Kimura

**Affiliations:** 1 Department of Hepatology, Tokyo Metropolitan Cancer and Infectious Diseases Center, Komagome Hospital, Tokyo, JPN

**Keywords:** chemotherapy response, hepatocellular carcinoma (hcc), non-b non-c juvenile hepatocellular carcinoma, antinuclear antibodies, alpha-fetoproteins

## Abstract

Hepatocellular carcinoma (HCC) usually occurs in settings of cirrhosis and chronic hepatitis B or C virus (HBV and HCV, respectively) infection; it is extremely rare in patients <40 years of age since viral- or alcohol-induced chronic hepatitis develops over a prolonged period. Juvenile HCC is mostly associated with persistent HBV infection; cases unrelated to HBV or HCV infection (non-B, non-C juvenile HCC) are sporadic and treated in the same way as classical HCC. A woman in her late 30s was diagnosed with HCC in a healthy liver; her imaging findings were typical of HCC with bone metastasis. She was administered a combination of tyrosine kinase inhibitors, immune checkpoint inhibitors, and vascular endothelial growth factor inhibitors. Throughout chemotherapy, the liver reserve was Grade A on the Child-Pugh classification and tumor markers remained under control without marked elevation. Our patient is the first reported long-term survivor of unresectable non-B, non-C juvenile HCC following chemotherapeutic treatment.

## Introduction

Hepatocellular carcinoma (HCC) often develops in patients with cirrhosis due to persistent hepatitis B virus (HBV) or hepatitis C virus (HCV) infection. Since HCC follows a gradual process (chronic hepatitis, followed by fibrosis, progression to cirrhosis, and eventual HCC development), it takes several decades to develop. Therefore, many HCC cases occur in patients older than 50 years [1]. Furthermore, almost all reported cases of HCC in younger ages have been associated with persistent HBV infection [2]. Non-B, non-C juvenile HCC is very rare and is treated in the same way as classical HCC that develops against a background of cirrhosis. The treatment for non-B, non-C juvenile HCC generally includes drug therapy, radiation therapy, resection, liver transplantation, and local ablation. Surgery is the main modality of treatment since it is often detected at an early stage. Conversely, systemic therapy is preferred when extrahepatic lesions are present and liver function is preserved. However, there are only a few reported cases of non-B, non-C juvenile HCC in which systemic treatment was considered as an option. Hence, the efficacy of chemotherapy for non-B, non-C juvenile HCC has not been clearly established.

Herein, we report a case of unresectable non-B, non-C juvenile HCC that responded to chemotherapy, and the patient achieved a long-term survival of 20 months.

## Case presentation

A healthy woman in her late 30s visited her local physician with back pain as the main complaint and was prescribed painkillers. Since her symptoms did not improve, she was re-examined. X-ray imaging showed osteolysis in the L3 vertebral body, and she was referred to our hospital for further examination in January 2020. She had no remarkable medical history and no family history of liver disease. However, her grandfather and grandmother had suffered from lung and stomach cancer, respectively. She had no history of smoking, drinking, or receiving any specific medications. She had never received a blood transfusion.　

**Figure 1:**
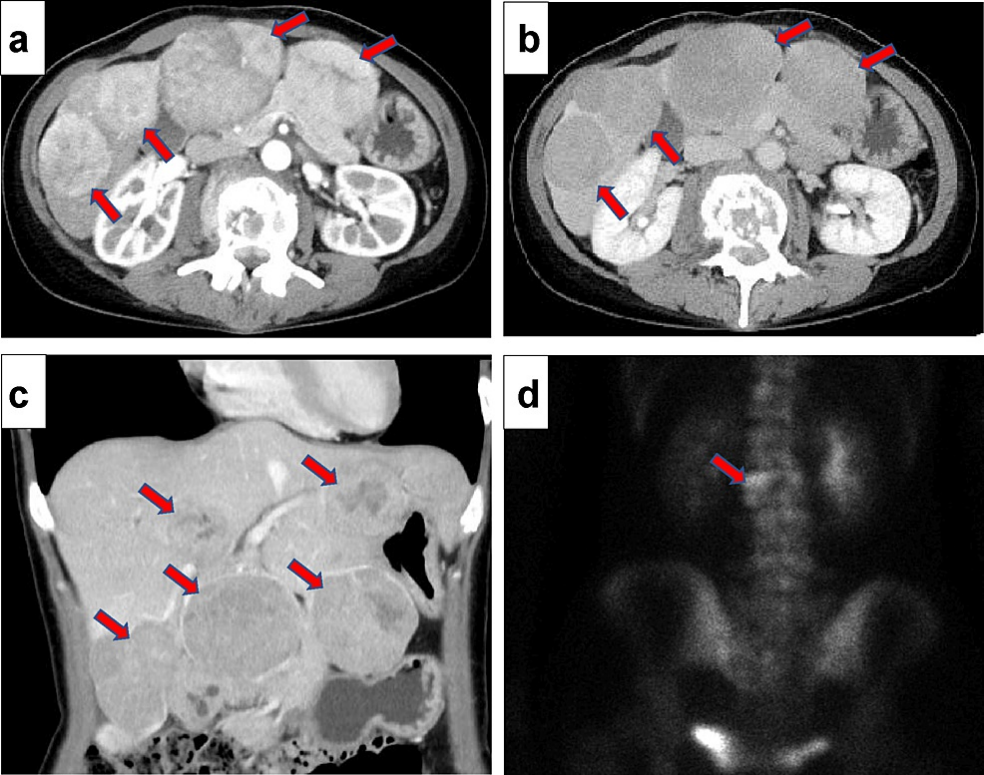
Diagnostic imaging of hepatocellular carcinoma: contrast-enhanced CT and bone scintigraphy findings a-c) Contrast-enhanced computed tomography shows that there are multiple tumors (red arrows) of up to 65 mm diameter in both lobes of the liver, which is a typical finding of hepatocellular carcinoma, with tumor enhancement in the early phase and washout in the late phase. d) Bone scintigraphy shows metastatic lesions (red arrows) in the L3 vertebral body.

Physical examination at our hospital revealed the following: height, 163 cm; weight, 51 kg; and body mass index, 19.2 kg/m^2^. Palpation of the abdomen revealed multiple masses without tenderness or edema. Laboratory blood test results on admission are shown in Table 1. The patient showed only mild hepatic dysfunction and well-preserved hepatic reserve. Several tumor markers were elevated, including alpha-fetoprotein (AFP) (458,500 ng/mL) and protein induced by vitamin K absence-II (PIVKA-Ⅱ) (5638 mAU/mL). Tests for HBV markers, such as hepatitis B surface antigen (HBsAg), hepatitis B surface antibody (HBsAb), and hepatitis B core antibody (HBcAb), showed negative results. Furthermore, serum HBV DNA was not detected. Tests for anti-HCV antibodies were also negative. Serum immunoglobulin (Ig)G and IgM levels were normal. In addition, tests for antinuclear and antimitochondrial antibodies were negative. Contrast-enhanced computed tomography (CT) showed multiple tumors of up to 65 mm in diameter in both lobes of the liver, a typical finding of HCC, with tumor enhancement in the early phase and washout in the late phase (Figure 1a-1c). Bone scintigraphy revealed metastatic lesions in the L3 vertebral body (Figure 1d).

**Table 1:** Laboratory blood examination on admission WBC: white blood cell, RBC: red blood cell, Hb: hemoglobin, HbA1c: glycated hemoglobin, Plt: platelet, PT-INR: prothrombin time-international normalized ratio, APTT: activated partial thromboplastin time, TP: total protein, BUN: blood urea nitrogen, Cre: creatinine, AST: aspartate aminotransferase, ALT: alanine aminotransferase, γ-GTP: γ-glutamyl transferase, ALP: alkaline phosphatase, T-Bil: total bilirubin, LDH: lactate dehydrogenase, CRP: C-reactive protein, AFP: alpha-fetoprotein, CEA: carcinoembryonic antigen, CA19-9: carbohydrate antigen 19-9, PIVKA-II: protein induced by vitamin K absence or antagonist II, HBsAg: hepatitis B surface antigen, HBsAb: hepatitis B surface antibody, HBcAb: hepatitis B core antibody, HCVAb: hepatitis C virus antibody, ANA: antinuclear antibody, AMA: anti-mitochondrial antibody, FreeT3: free triiodothyronine, FreeT4: free thyroxine, TSH: thyroid stimulating hormone, IgG: immunoglobulin G, IgA: immunoglobulin A, IgM: immunoglobulin M

Parameter	Value	Parameter	Value	Parameter	Value
WBC	5000/μL	Na	139 mmol/L	AFP	458,500 ng/mL
RBC	415 × 10^4^/μL	K	3.9 mmol/L	PIVKA-Ⅱ	5683 mAU/mL
Hb	11.8 g/dL	CL	99 mmol/L	CEA	1.5 ng/mL
Plt	46.1 × 10^4^/μL	Glu	102 mg/dL	CA19-9	8.8 U/mL
PT-INR	1.06 s	HbA1c	5.2%	HBsAg	(-)
APTT	27.6	CRP	0.32 mg/dL	HBsAb	(-)
TP	7.4 g/dL	Fe	91 ng/mL	HBcAb	(-)
Albumin	4.1 g/dL	Cu	141μg/dL	HBV-DNA	(-)
BUN	11 mg/dL	FreeT3	2.48 pg/mL	HCVAb	(-)
Cre	0.62 mg/dL	FreeT4	1.22 ng/dL	HCV-RNA	(-)
AST	36 U/L	TSH	1.57μIU/mL		
ALT	28 U/L	IgG	1221 mg/dL		
LDH	203 U/L	IgA	400 mg/dL		
ALP	231 U/L	IgM	145 mg/dL		
γ-GTP	163 U/L	AMA	<20		
T-Bil	0.5 mg/dL	ANA	<40		

**Figure 2:**
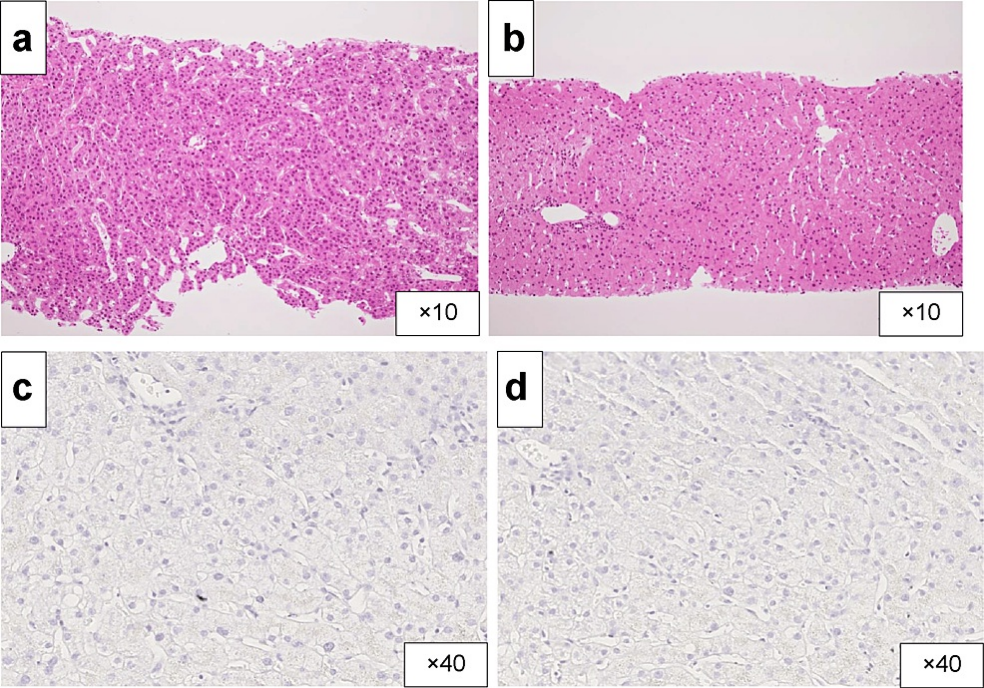
Histopathological and immunohistochemical analysis of a liver tumor biopsy revealing hepatocellular carcinoma a-b) A liver tumor biopsy was performed to confirm the diagnosis. Haematoxylin and eosin staining of the carcinomatous specimen shows well-differentiated hepatocellular carcinoma and that of noncancerous portions shows no fibrosis and/or inflammatory infiltration. c-d) Immunostaining for hepatitis B surface and hepatitis B core antibodies showed negative results.

Since the patient with suspected HCC was young and did not have cirrhosis, a liver tumor biopsy was performed with a 16-gauge automatic needle to confirm the diagnosis. The biopsy showed well-differentiated HCC tumor morphology, while immunostaining for HBsAb and HBcAb showed negative results (Figure 2). Consequently, the patient was diagnosed with cT3N0M1 stage IV HCC (according to the Union for International Cancer Control Seventh Edition classification).

**Figure 3:**
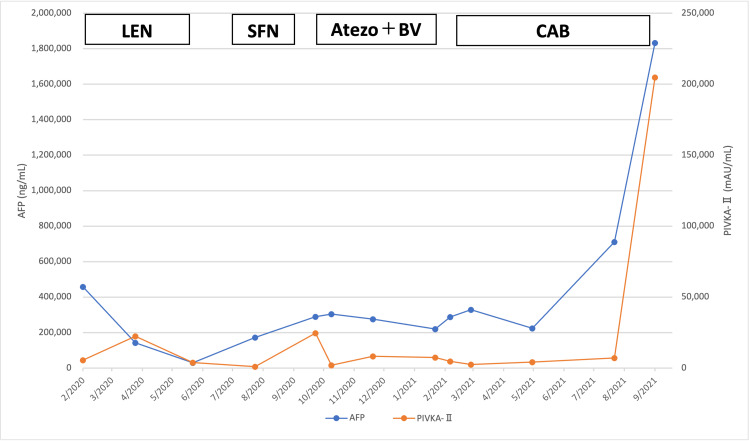
Juvenile hepatocellular carcinoma: treatment course and variations in tumor markers AFP, alpha-fetoprotein; PIVKA-II, protein induced by vitamin K absence-II; Atezo, atezolizumab; BV, bevacizumab; CAB, cabozantinib; LEN, lenvatinib; SFN, sorafenib

**Figure 4:**
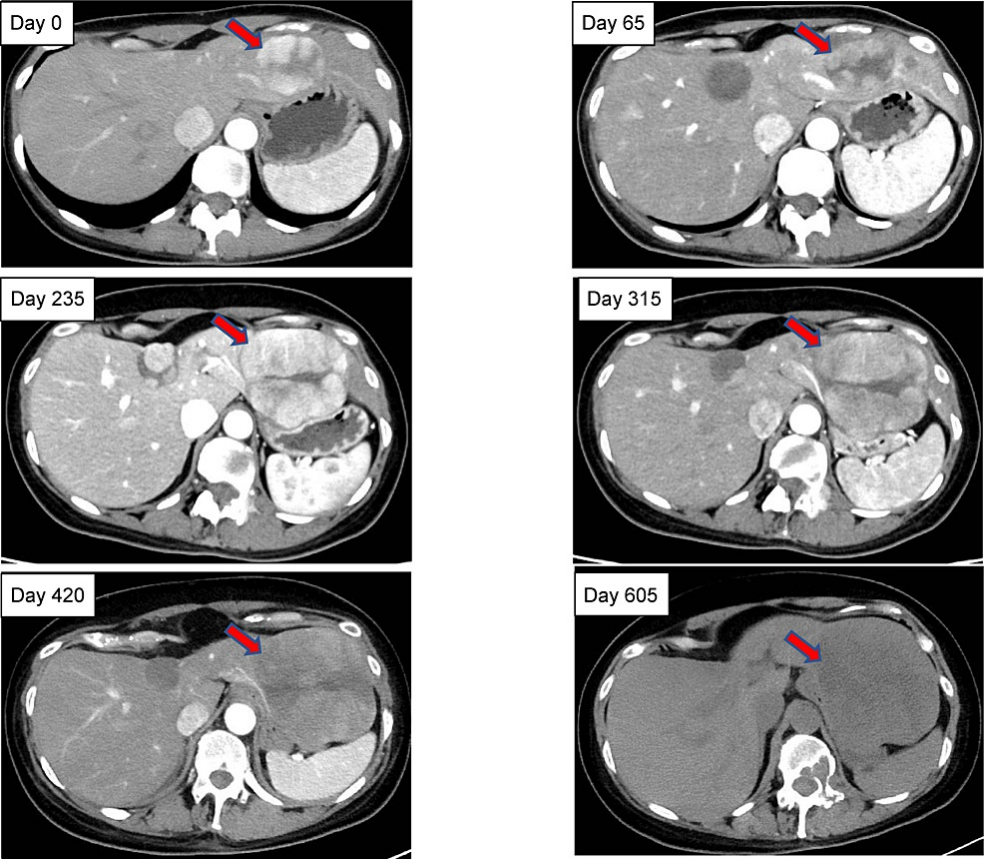
Sequential changes in liver tumor size in the left lobe following treatment with lenvatinib Time series of tumor changes in the left lobe of the liver (red arrows). The initial treatment with lenvatinib had an inhibitory effect; thereafter, a gradual trend toward enlargement was observed.

Palliative radiation therapy (20 Gy/5fr) for metastatic lesions was initiated on February 6, 2020 (designated as day 0), followed by a multi-target tyrosine kinase inhibitor (TKI) and lenvatinib (LEN, 8 mg/day). Although her symptoms improved, LEN was discontinued on day 113 because of anorexia and hypertension and was replaced by another multi-target TKI, sorafenib (SFN), on day 160. SFN was started at 400 mg/day and discontinued on day 235 for progressive disease (PD) evaluation by CT. Combination therapy with atezolizumab (Atezo) and bevacizumab (BV) was initiated on day 250 and continued until day 420, when PD evaluation by CT was performed. Atezo and BV were administered at 1200 mg/dose and 15 mg/kg, respectively, once every three weeks. Cabozantinib (CAB) was initiated at 60 mg/day on day 440; however, it was discontinued on day 605 due to the patient’s deteriorating health condition, caused by bone metastases-related fractures, and she was placed on palliative treatment. The liver reserve remained good, with Grade A on the Child-Pugh classification, throughout the treatment course. The treatment course and tumor marker changes are shown in Figure 3. Figure 4 shows a time series of CT images of the tumor in the left lobe of the liver. The initial LEN treatment had an inhibitory effect; thereafter, a gradual trend toward enlargement was observed. Unfortunately, the patient expired on October 20, 2021 (day 622) due to a ruptured HCC (Figure 3).

## Discussion

In general, persistent chronic inflammation leads to fibrosis and cirrhosis, which often culminates in HCC. The main causes of cirrhosis are persistent HBV and HCV infection and alcohol intake, which accounts for approximately 80% of all patients with HCC. HCC underlying cirrhosis caused by HBV and HCV is widely recognized, especially in Asia. In addition, the prevalence of HCC underlying cirrhosis due to alcoholic liver disease or non-alcoholic fatty liver disease (NAFLD) has reportedly increased over the past few decades [3]. In areas with a high prevalence of HBV-associated cirrhosis, the cumulative risk of HCC over five years is 15%, while in Western regions, it stands at 10% [4]. The cumulative HCC incidence rates among patients with alcoholic liver disease with compensated and decompensated cirrhosis are 6.8% and 37.4%, respectively, at 10 years [5]. By contrast, a systematic review on the cumulative HCC incidence rate in patients with NAFLD or non-alcoholic steatohepatitis (NASH), among which few or none have cirrhosis, indicated minimal HCC risk, with mortality ranging from 0% to 3% over a period of up to 20 years [6]. For patients with NASH and cirrhosis, the cumulative HCC rates range from 2.4% over seven years to 12.8% over three years [6]. However, a small fraction of HCC cases occur in the absence of cirrhosis. Although nearly 89% of juvenile HCC cases are purportedly associated with persistent HBV infection [2], the incidence of non-B, non-C HCC due to NASH and other causes has been increasingly reported in recent times, with the incidence of juvenile HCC in patients younger than 40 years at approximately 0.8%.

The diagnosis of non-B, non-C juvenile HCC is generally made in the same manner as that for classical HCC that develops on a background of cirrhosis; however, it is important to differentiate it from other liver diseases since it occurs at a young age. Fibrolamellar HCC (FLC) is a special type of liver cancer that develops in young individuals independent of hepatitis and cirrhosis [7] and is characterized by the presence of a large tumor with calcification and a central scar. The exact frequency of non-B, non-C juvenile HCC in a healthy liver is unknown because of the limited number of published case reports. Known risk factors and characteristics of nonviral noncirrhotic HCC include obesity, type 2 diabetes, alcohol consumption, and genetic factors, especially mutations in HCC-related genes, such as TERT, TP53, and CTNNB1 [8,9]. Non-B, non-C juvenile HCC has not yet been adequately studied, and guidelines for its treatment have not been established. Hence, the treatment regimen generally follows those used for classical HCC, that is, surgery and transcatheter arterial embolization (TAE). The present case involved a woman in her late 30s who tested negative for all hepatitis virus-related markers, such as HBsAg, HBsAb, HBcAb, and HCVAb, and neither HBV DNA nor HCV RNA was detected. Pathological examination of the liver biopsy specimen showed no findings suggestive of chronic hepatitis or cirrhosis. These pathological findings were also different from those of FLC. Since its publication in 2018, the Barcelona Clinic Liver Cancer (BCLC) prognosis and treatment strategy is generally used to determine the treatment plan for HCC [10]. According to the BCLC prognosis and treatment strategy, the treatment of choice for advanced stage C patients with extrahepatic lesions, preserved liver function, and a performance status of 1-2 is systemic therapy. As our patient was diagnosed with cT3N0M1 stage IV HCC, chemotherapy was selected. The revised RECIST guideline (version 1.1) was used to evaluate the response [11].

A rigorous search in PubMed yielded only eight reported cases of non-B, non-C juvenile HCC without chronic hepatitis or cirrhosis, including the present case (Table 2). Among these, only three cases (37.5%) were associated with extrahepatic lesions at the time of diagnosis. The majority of cases were indicated for surgery and TAE; those who were not indicated for surgery and TAE showed a poor prognosis. Recently, new molecular-targeted agents and immune checkpoint inhibitors have become available for the treatment of HCC. The present case was successfully treated with molecular-targeted agents, such as LEN, SFN, and CAB, and combination therapy with immune checkpoint inhibitors and molecular-targeted agents, such as Atezo and BV. Considering that the average survival time for patients with stage IV well-differentiated HCC is generally less than one year, yet our patient survived for almost two years, chemotherapy was considered effective in the present case of non-B, non-C juvenile HCC. This may be due to the fact that the patient had preserved liver reserve. However, our patient had multiple bone metastases, and combination therapies such as those used in the present case require appropriate patient selection and management of side effects. Thus, combinational therapy using multiple therapeutic agents with wide-ranging effects may achieve long-term survival, which may be difficult to achieve with conventional therapies.

**Table 2:** Non-B, non-C juvenile hepatocellular carcinoma: previous case reports F, female; M, male; AFP, alpha-fetoprotein; PIVKA-II, protein induced by vitamin K absence-II; TAE, transcatheter arterial embolization; ND, not described

No.	Reference	Author	Year	Age (years)	Sex	Extrahepatic metastasis	AFP	PIVKA-Ⅱ	Treatment	Follow-up period (month)	Outcome
1	[12]	Fujita et al.	1995	25	F	-	35,200	14.4	TAE	11	Alive
2	[13]	Sato et al.	1996	23	F	-	10,320	Normal	TAE	24	Deceased
3	[14]	Tanaka et al.	1996	39	F	-	Normal	Normal	Surgery	ND	ND
4	[15]	Assed et al.	2009	26	F	Lung	>300	ND	Palliative chemotherapy	3	Deceased
5	[16]	Fukui et al.	2011	20	M	-	182	75,000	Surgery	36	Alive
6	[17]	Hashimoto et al.	2021	20's	M	Lung	76,860	74,849	Chemotherapy	9	Deceased
7	[18]	Onishi et al.	2022	23	F	-	941.8	1170	Surgery	60	Alive
8	Present case report	Kimura et al.		39	F	Bone	45,850	5683	Chemotherapy	20	Deceased

## Conclusions

This case exemplifies the effectiveness of systemic chemotherapy in a patient with non-B, non-C juvenile HCC, notably in the context of a well-preserved liver function. Despite the presence of bone metastases, which typically necessitates systemic chemotherapy as a standard treatment option, the preserved hepatic reserve in this case enabled the aggressive trial of multiple treatment regimens. This approach led to an extended survival, suggesting that in non-traditional cases of HCC, expanding the spectrum of treatment options and tailoring strategies to individual patient profiles can be crucial. This case underscores the importance of personalized treatment plans, especially in young patients with good liver function, highlighting the potential for improved outcomes even in the face of advanced diseases.
